# The Australian Curriculum over a decade: the status of the promises, problems, and possibilities

**DOI:** 10.1007/s41297-022-00171-x

**Published:** 2022-10-18

**Authors:** Deborah Price

**Affiliations:** Centre for Research in Educational and Social Inclusion (CRESI), University of South Australia, Adelaide, Australia

**Keywords:** Australian curriculum, Curriculum studies, Inclusive curriculum

Over a decade has passed since Australia’s first national curriculum, the Australian Curriculum (AC), was implemented in 2011. The development was reported as ‘unprecedented in Australia’s history, with the processes involved having produced unprecedented opportunities for debates and position-taking in Australia on the curriculum for the twenty-first century’ (Yates, 2018, p. 137). The development and initial versions of the official AC were documented and debated by 21 leading curriculum scholars in the Australian Curriculum Studies Association publication, ‘The Australian Curriculum: Promises, Problems, and Possibilities’ (Reid & Price, 2018). The edited collection aimed to record the background and rationale of the AC and to ‘contribute to insights about the processes of curriculum making in a federal system… and assist people to understand and analyse the curriculum debates’ (Reid & Price, 2018, p. v). In efforts to provide a responsive and relevant national curriculum, continuous development has ensued across updated versions, with the latest version 9.0, released in 2022. Thereby, this point and counterpoint engages with three Australian scholars in critiquing the progress towards meeting the intended aspirations and educational goals for young Australians across the fields of; *The Arts and Cross-Curriculum Priorities (CCP)*, *Science, Technologies, Engineering, and Mathematics (STEM),* and *English*. A key theme emerging across the papers reinforces the ‘recognition that a quality curriculum is always in a state of becoming’ (Reid and Price 2018, p. viii) and the need for curriculum to be dynamic, living, and responsive.

## The status of curriculum studies in Australia

As Yates (2018) highlighted, Australian writers, are highly astute in curriculum studies, including the interplay between policy contexts, debates, and mechanisms, as well as how educators engage with the curriculum and international curriculum movements. She asserts how this is ‘not a combination that necessarily characterises curriculum inquiry in other countries’ (Yates, 2018, p. 138). The matrix structure of the AC ‘combining “learning areas” (or subject-based forms of knowledge); general capabilities; and “cross-curriculum priorities” was one way of bringing together some of the different ways of thinking about what school curriculum should represent, and what elements are important today’ (Yates, 2018, p. 137).

As the curriculum has developed across the subsequent versions, achievement of the aims underpinning the official rationale for moving from the state-based to the national AC continues to be debated. As Reid (2018) documented, the rationale included: ‘more uniformity and consistency… prevent duplication of resources’ and reducing ‘variations in retention rates and student achievement’ (p. 15). Notwithstanding the variations of implementation of the AC across jurisdictions and debates regarding the educational engagement and retention of young people in formal education, there also continue to be questions regarding the nature of the curriculum. These span across early years to senior secondary education and teacher training, including: questioning what is valued in relation to knowledge and what constitutes learning; considerations regarding the cultural foundation of the curriculum and equity and inclusion; and who determines what is prioritised in the curriculum. These debates continue to be influenced by a range of factors including; the predominant prioritisation of international and national performativity and accountability measures in select learning areas within international, national and local assessments, the COVID-19 pandemic, and the status of the teacher workforce including retention and wellbeing. Additional factors include the introduction of national professional standards for teachers and initial teacher education programs and the ongoing requirement for professional development for practising educators.

The AC intended curriculum advocates for ‘setting the expectations for what all young Australians should be taught, regardless of their background or where they live’ (ACARA 2022a). As such, Brennan and Zipin’s (2018) call for doing ‘curriculum justly’ continues to resonate within the latest AC development, to ensure that within the curriculum, the ‘most marginalised are recognised and representatively empowered, diverse traditions of knowledge and action are valued, and spaces are opened for meaningful local and global content’ (p. 186). In continuing to shape a national curriculum inclusive of diverse learners (Price & Slee, 2018, 2021), each version of the AC continues to take steps to address these challenges, with version 9.0 ‘a more stripped-back and teachable curriculum that identifies the essential content of our children should learn… reduction in content… better alignment between achievement standards and content descriptions, and improved links between learning areas and the general capabilities and cross-curriculum priorities’ (ACARA 2022b). Yet what is deemed as essential content continues to be contested, which is necessary for the aspiration to fully ‘include all learners in education, and to respect, value and apply diverse knowledge, capabilities, and lived experiences as the foundation for learning and achievement’ (Price & Slee, 2018, p. 211). Within version 9.0, key areas intended for change have focused on ‘deepening students’ understanding of First Nations Australian histories and cultures, the impact on—and perspectives of—First Nations Australians of the arrival of British settlers as well as their contribution to the building of modern Australia’ as well as promoting respectful relationships and ‘strengthening and making explicit teaching about the origins and Christian and Western heritage of Australia’s democracy, as well as about the diversity of Australian communities’ (ACARA 2022b).

## Reflections on the status of the arts and cross-curriculum priorities (CCP), STEM, and English

The first paper presented in this point and counterpoint is presented by Belinda MacGill, titled: *The Australian Curriculum, Assessment, and Reporting Authority (ACARA). Holding responsibility: The Arts Curriculum and the Cross-Curriculum Priorities.* In this reflective piece, the reader is reminded of ACARA’s invitation for teachers to integrate the CCPs: Aboriginal and Torres Strait Islander Histories and Knowledges, Sustainability and Asia, and Australia’s Engagement with Asia, into the arts curriculum. MacGill identifies the socio-historical and political responsibility in prioritising CCP as core to the AC and that the art curriculum can play a significant role. Yet, the responsibility of such important educational work is suggested to be primarily held by teachers who need the space for deep learning regarding epistemologies and ontologies existing within the disciplinary fields of the CCPs. This paper draws attention to the ‘problematics of a national curriculum and the sub-text of responsibility’.

The second paper by Simon Leonard: *The Arrival of STEM in the Science and Mathematics Curriculum Increases the Epistemic Demands on Teachers* acknowledges the initial reference to STEM in version 9.0 of the AC. Leonard identifies the unusual pathway of STEM into the curriculum and the tension of curriculum development between the traditional disciplinary knowledge and skills needed for higher education and scientific careers and the development of scientific and mathematical understandings and dispositions for young people’s societal participation. As STEM continues to emerge in the AC, Leonard cautions how it must be seen within the wider discourse aligning ‘the sciences with the needs of a specific image of future industry’.

The third paper is presented by Jenni Carter: *Stories that Matter: Engaging Students for a Complex World Through the English Curriculum.* Carter shares some of the developments within English and the framework that the AC provides for engaging children and young people with stories that provide pleasure and understanding of living in and sharing worlds. The key tenet of this paper centres on the ‘How we teach and learn about stories matters’, calling for curriculum development to continue to engage with the contested complexities and paradoxes with an understanding of the significant ‘role of language as an individual, social, cultural, and political practice’.

## References

[CR1] Australian Curriculum Assessment and Reporting Authority (ACARA) (2022a). Version 9.0 The Australian Curriculum. Retrieved August 26, 2022 from https://v9.australiancurriculum.edu.au/

[CR2] Australian Curriculum Assessment and Reporting Authority (ACARA) (2022b). What’s changed in the new Australian Curriculum? Retrieved August 26, 2022 from https://v9.australiancurriculum.edu.au/resources/stories/curriculum-changes

[CR3] Brennan, M. & Zipin, L. (2018). Curriculum for all? Exploring potentials for [in]justice in the Australian Curriculum. In A. Reid & D. Price (Eds.), *The Australian Curriculum: Promises, Problems and Possibilities*. Australian Curriculum Studies Association, Canberra. ISBN-13: 978-1-875864-77-5.

[CR4] Price, D., & Slee, R. (2018). An Australian Curriculum that includes diverse learners: The case of students with disability (Chapter 17, pp. 211–226). In A. Reid & D. Price (Eds.), *The Australian Curriculum: Promises, Problems and Possibilities*. Australian Curriculum Studies Association, Canberra. ISBN-13: 978-1-875864-77-5.

[CR5] Price D, Slee R (2021). An Australian Curriculum that includes diverse learners: The case of students with disability. Curriculum Perspectives.

[CR6] Reid, A. & Price, D. (Eds.). (2018). *The Australian Curriculum: Promises, problems and possibilities*. ACT: Australian Curriculum Studies Association, Canberra. ISBN-13: 978-1-875864-77-5.

[CR7] Reid, A. (2018). The journey towards the first Australian Curriculum (Chapter 1, pp. 3–18). In A. Reid & D. Price (Eds.), *The Australian Curriculum: Promises, problems and possibilities.* Australian Curriculum Studies Association, Canberra. ISBN-13: 978-1-875864-77-5.

[CR8] Yates L (2018). The curriculum conversation in Australia. Curriculum Perspectives.

